# Nine changes needed to deliver a radical transformation in biodiversity measurement

**DOI:** 10.1073/pnas.2519345123

**Published:** 2026-03-04

**Authors:** William J. Sutherland, Neil D. Burgess, Scott V. Edwards, Julia P. G. Jones, Pamela S. Soltis, David Tilman, Julie M. Allen, Herizo T. Andrianandrasana, Cathrine J. Armour, Tom August, Kamaljit S. Bawa, Sallie Bailey, Tanya Birch, Philipp H. Boersch-Supan, Jeannine Cavender-Bares, Mark Blaxter, Rebecca Chaplin-Kramer, Barnabas H. Daru, Adriana De Palma, Cristina Eisenberg, Chris S. Elphick, Robert P. Freckleton, Winifred F. Frick, Andrew Gonzalez, Scott J. Goetz, Lior Greenspoon, Christina M. Grozingeree, Don L. Hankins, Jonny Hazell, Nick J. B. Isaac, Marco Lambertini, Harris A. Lewin, Oisin Mac Aodha, Anil Madhavapeddy, EJ Milner-Gulland, Ron Milo, James O’Dwyer, Andy Purvis, Nick Salafsky, Heather Tallis, Iroro Tanshi, Varsha Vijay, Martin Wikelski, David R. Williams, S. Hollis Woodard, Gene E. Robinson

**Affiliations:** ^a^Conservation Science Group, Department of Zoology, University of Cambridge, Cambridge CB2 3QZ, United Kingdom; ^b^United Nations Environment Programme World Conservation Monitoring Center, Cambridge CB3 0DL, United Kingdom; ^c^Center for Macroecology, Evolution and Climate, GLOBE Institute, University of Copenhagen, Copenhagen 1350, Denmark; ^d^Department of Organismic and Evolutionary Biology, Harvard University, Cambridge, MA 02138; ^e^School of Environmental and Natural Sciences, Bangor University, Bangor LL57 2UW, United Kingdom; ^f^Institute of Environmental Biology, Utrecht University, Utrecht 3584 CH, The Netherlands; ^g^University of Florida Biodiversity Institute, University of Florida, Gainesville, FL 32611; ^h^College of Biological Sciences, University of Minnesota, St. Pail, MN 55108; ^i^Department of Biological Sciences, Virginia Tech, Blacksburg, VA 24060; ^j^Department of Geosciences and Geography, University of Helsinki, Helsinki FI-00014, Finland; ^k^Taskforce on Nature-related Financial Disclosures, Green Finance Institute, London EC2A 2DQ, United Kingdom; ^l^United Kingdom Centre for Ecology and Hydrology, Crowmarsh Gifford, Wallingford OX10 8BB, United Kingdom; ^m^Department of Biology, University of Massachusetts, Boston, MA 02125; ^n^Ashoka Trust for Research in Ecology and the Environment, Bengaluru 560064, India; ^o^Natural England, Lancaster House, Hampshire Court, Newcastle upon Tyne NE4 7YH, United Kingdom; ^p^Google Geo Sustainability, Google Limited Liability Company, Mountain View, CA 94043; ^q^British Trust for Ornithology, The Nunnery, Thetford, Norfolk IP24 2PU, United Kingdom; ^r^Tree of Life Programme, Wellcome Sanger Institute, Hinxton, Cambridgeshire CB10 1SA, United Kingdom; ^s^Global Science, World Wide Fund for Nature, San Francisco, CA 94105; ^t^Department of Biology, Stanford University, Stanford, CA 94305; ^u^Biodiversity Futures Lab, Natural History Museum, London SW7 5BD, United Kingdom; ^v^Ecocultural Resources, Corvallis, OR 97330; ^w^Department of Ecology and Evolutionary Biology and Center of Biological Risk, University of Connecticut, Storrs, CT 06269; ^x^School of Biosciences, University of Sheffield, Sheffield S10 2TN, United Kingdom; ^y^Bat Conservation International, Austin, TX 78746; ^z^Ecology and Evolutionary Biology, University of California Santa Cruz, Santa Cruz, CA 95060; ^aa^Department of Biology, McGill University, Montreal, QC H3A 1B1, Canada; ^bb^School of Informatics and Computing, Northern Arizona University, Flagstaff, AZ 86011; ^cc^Department of Plant and Environmental Sciences, Weizmann Institute of Science, Rehovot 76100, Israel; ^dd^Department of Entomology, Center for Pollinator Research, Huck Institutes of the Life Sciences, University Park, PA 16803; ^ee^Geography and Environmental Studies, California State University, Chicago, CA 95929; ^ff^The Royal Society, London SW1Y 5AG, United Kingdom; ^gg^Nature Positive Initiative Secretariat, Trelex 1270, Switzerland; ^hh^Julie Ann Wrigley Global Futures Laboratory and College of Global Futures Arizona State University, Tempe, AZ 85287; ^ii^School of Informatics, University of Edinburgh, Edinburgh EH8 9AB, United Kingdom; ^jj^Department of Computer Science and Technology, University of Cambridge, Cambridge CB30FD, United Kingdom; ^kk^Department of Biology, University of Oxford, Oxford OX1 3SJ, United Kingdom; ^ll^Department of Plant Biology and Carl R. Woese Institute for Genomic Biology, University of Illinois, Urbana, IL 61801; ^mm^Foundations of Success, Bethesda, MD 20816; ^nn^Center for Coastal Climate Resilience, University of California Santa Cruz, Santa Cruz, CA 95060; ^oo^Department of Biology, University of Washington, Seattle, WA 98195; ^pp^Science Based Targets Network, New York, NY 10036; ^qq^Department of Migration, Max Planck Institute of Animal Behavior, Radolfzell 78315, Germany; ^rr^Department of Biology, University of Konstanz, Konstanz 78457, Germany; ^ss^Sustainability Research Institute, University of Leeds, Leeds LS2 9JT, United Kingdom; ^tt^Department of Entomology, University of California, Riverside, CA 92506; ^uu^Department of Entomology, University of Illinois, Urbana, IL 61801

**Keywords:** AI, eDNA, auditory data, image recognition, Indigenous Knowledge

## Abstract

Biodiversity is declining in many parts of the world. Biological diversity measurement and monitoring are fundamental to the assessment of the causes and consequences of environmental changes, identification of key areas for the protection of biodiversity or ecosystem services, determining the effectiveness of actions, and the creation of decision-support tools critical to maintaining a sustainable planet. Biodiversity measurement is rapidly changing due to advances in citizen science, image recognition, acoustic monitoring, environmental DNA, genomics, remote sensing, and AI. In this perspective, we outline the exciting opportunities these developments offer but also consider the challenges. Our key recommendations are to 1) Capitalize on the ability of novel technology to integrate data sources 2) agree to standard methods for data collection 3) ensure new technologies are calibrated with existing data; 4) fill data gaps by using emerging technologies and increasing capacity, especially in the tropics; 5) create living safeguarded databases of trusted information to reduce the risk of poisoning by AI hallucinated, or false, information; 6) ensure data generation is valued; 7) ensure respectful incorporation of Indigenous Knowledge; 8) ensure measurements enable the quantification of effectiveness of actions, and 9) increase the resilience of global datasets to technical and societal change. Radical new collaborations are needed between computer scientists, engineers, molecular biologists, data scientists, field ecologists, citizen scientists, Indigenous peoples, policymakers, and local communities to create the rigorous, resilient, accessible biodiversity information systems required to underpin policies and practices that ensure the maintenance and restoration of ecological systems.

Biodiversity—a major determinant of ecosystem productivity, stability, and resilience—delivers a wide range of benefits to society (1–3). Yet, as shown by the summary of key numbers in Fig. 1 biodiversity is in rapid and widespread decline in the face of anthropogenic pressures, driving a range of serious problems for society (30). Accurate and reliable biodiversity measurement is fundamental to the conservation, restoration, and stewardship of nature and maintaining benefits from ecosystems. These measurements underpin the delivery of policies and practices that provide necessary change to ensure nature’s resilience. Specifically, measurement is foundational to our knowledge of global patterns, including measuring the changes in populations, communities and ecosystems, the nature and magnitude of threats, the effectiveness of potential solutions, and the progress toward conservation targets.

**Fig. 1.**
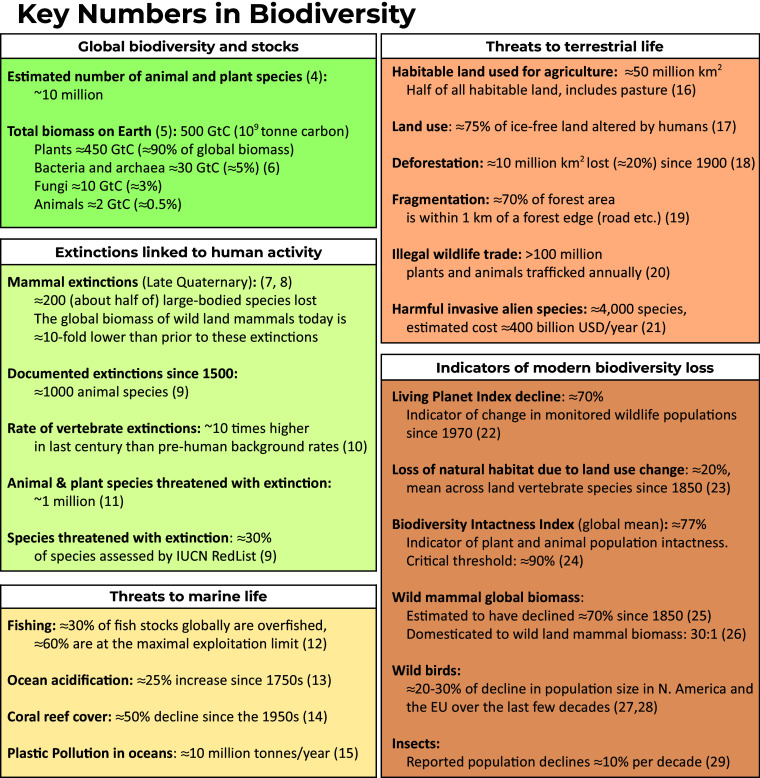
Key Numbers in Biodiversity. A summary of key numbers measuring the current state of biodiversity globally. We report highlights from the global quantification of this inherently multifaceted and heterogenous concept and phenomena. The number of significant digits used tried to reflect the variation in expert opinion and references.

Biodiversity measurement is undergoing rapid change, especially through citizen science, image recognition, acoustic monitoring, environmental DNA (eDNA), genomics, remote sensing, and AI. These changes bring opportunities through increasing the scale and resolution of biodiversity measurement. However, they also bring challenges including ethical concerns surrounding the risks of biodiversity monitoring technology undermining privacy (31), and unequal access to fast-moving technology exacerbating existing inequalities and power imbalances (32, 33).

There are currently greater demands for biodiversity data than ever before. Businesses increasingly need to measure and manage their impact and dependencies on biodiversity, driven by legislation (for example the EU’s Corporate Social Responsibility Directive and Deforestation Regulation) and voluntary compliance with the Taskforce on Nature-related Financial Disclosures, the Science Based Targets Network, or similar initiatives, driven by pressure from consumers (34). There is also an explosion of interest in measuring the effectiveness of conservation policies and practices to inform truly evidence- based decision-making (35), including recognition of the need to design evaluation into conservation actions (36, 37). For governments, a major demand is monitoring progress toward their own national legislation, such as England’s Environment Act, which includes targets to halt and reverse biodiversity loss, or commitments to international agreements, such as Kunming-Montreal Global Biodiversity Framework (38).

This combination of dramatic advances in technology for measuring biodiversity, alongside unprecedented societal demand, creates an extraordinary opportunity to transform evidence-based biodiversity policy and practice. In this perspective, we first introduce the scope of emerging technologies and the flood of new data being generated. We then suggest nine recommendations for ensuring biodiversity measurement can fulfill its potential in helping to address the global biodiversity crisis.

## A Flood of New Data from Emerging Technologies

There has been a remarkable explosion in the amount of biodiversity data available (Table 1), reflecting the proliferation of new technologies for observation and a culture shift toward data sharing. The scale of the flood of data is illustrated by the Global Biodiversity Information Facility (GBIF), which manages a vast source of species occurrence data and is adding records at a rate exceeding 420 million a year. GBIF is now integrating data from museums and herbaria, eDNA, citizen science like eBird, and data from Environmental Impact Assessments. These disparate data types are brought together within a central database and then made available via to anyone who wants to use it. Over 10,000 scientific papers have made use of GBIF mediated data, and it is being used widely by governments for various purposes.

**Table 1.** Some types of biodiversity data and their coverage

Type of data	Examples of extent
Atlases	Cover a range of taxa including over 600 bird atlases across 93 countries, with over 380,000 participants. The Xerces Society bumble bee atlas covers 20 US states, and with >110,000 records from >4,000 participants.
Auditory	Xeno-canto is approaching 1 million wildlife sound recordings (mostly birds); Macaulay Library (Cornell) has 2.8 million bird recordings, and is growing at an annual rate of over 15%.
Camera traps	A number of camera trap platforms streamline data processing. The Wildlife Insights platform has over 200 million images from almost 3,000 projects and includes over 4,000 species.
Citizen science	In June 2025 eBird passed 2 billion observations from over 14 million checklists and 1.1 million eBird participants.
Citizen science with standard processes	Many large monitoring schemes (North American Breeding Bird Survey, Pan-European Common Bird Monitoring Scheme, Southern African Bird Atlas Project, European Butterfly Monitoring Scheme, Snapshot USA, Snapshot Europe, etc) have a rigorous process.
Flora	Thousands of flora, covering local, national and broader regions, are published that list species, describe habitat and their status.
DNA marker data	The Barcode of Life database collates marker gene information from over 20 million specimens corresponding to approximately 1.3 million species. The database is growing rapidly with contributions from around the globe.
Genomes	Earth Biogenome Project plans to generate high-quality reference genomes for 10,000 unique species before the end of 2027, and 150,000 by 2031. As of June 2025, data for 3,956 species was publicly available.
Herbaria	Index Herbariorum lists 3,567 herbaria worldwide containing 396 million specimens. Many are digitising specimens, which are often then made available online.
Image recognition	iNaturalist has 248 million observations supported by photographs taken by citizen scientists, who may be aided by AI identification, with 155 million being verified by the community.
Indigenous and local knowledge	PISUNA (Piniakkanik Sumiiffinni Nalunaarsuineq) has 1,052 items of Inuit knowledge on animal abundance, distributions, condition and behaviour and ice conditions.
Local and national data centres	These occur in at least a hundred countries with data sources that often overlap the others included here.
Museums	Museums globally contain about 3 billion specimens with digitisation, including with AI, underway at many institutions.
Tracking data	MOVEBANK has 8 billion locations of 1,546 taxa from 9,113 studies.
Remote sensing	1000+ datasets are provided in the Google Earth Engine Data Catalog.

Robotic and autonomous systems are likely to change many of the technologies described in this section as they deliver sampling, for example, through robots or drones (39). eDNA, the DNA released into the environment by organisms, can be detected from sampling soil, water, or air, enabling the cost-effective identification of species present. It has been widely used in targeted species detection studies with PCR and qPCR assays, and in community (i.e., multispecies) studies using metabarcoding (40). Researchers can compare communities, including microbial communities (which respond especially quickly to changes in conditions), through sampling of preserved samples, such as in permafrost, lake sediments, peat, or air samples (41) to examine changes over long periods of time. For example, Kjær et al. examined a 2-My-old deposit in Greenland identifying 102 genera from the eDNA, including determining the habitat, identifying many plants missed from macrofossil and pollen recording, and showing the presence of mastodons, reindeer, rodents, and geese, all ancestral to their present-day and late Pleistocene relatives (42). eDNA is efficient for monitoring current ecosystems, especially from water or soil samples, or for monitoring species at scale (43). The utility of the approach for a given taxonomic group in a geographic area depends on the availability of appropriate reference DNA libraries, and this is rapidly increasing.

As well as the ability to identify species based on fragments of eDNA, rapid advances in sequencing technology has enabled the generation of high-quality complete genomes(including reference genomes), which allow species identification, the understanding of species’ genetic diversity and the distribution of variants in space and time (44) as well as the species’ history. This information can be used to identify genotypes that are more resilient for conservation/assisted migrations and populations that are experiencing stress.

Image and acoustic signal recognition, the ability for AI to identify species based on their appearance or the sounds they make, are progressing fast toward large-scale automated data collection, for example, from distributed networks of acoustic sensors or camera traps. Automated identification of auditory recordings is rapidly improving both for species on land and in the oceans (45). For example, in 2021 Kahl et al. reported that BirdNET, using deep artificial neural networks, could identify almost a thousand bird species and replicate seasonal detection patterns obtained by human observers (46). By 2025, the number of species BirdNET could identify had trebled (https://birdnet.cornell.edu/). Similar methods applied to frogs from the Philippines show both high recognition accuracy and the capacity to detect species previously undescribed by scientists (47). Improved methods development, the rapid accumulation of recordings that can be used for model training, and the nascent creation of benchmarked datasets for taxa ranging from insects (48) to whales (49) suggests that the use and sophistication of these methods will grow rapidly.

Citizen science is another area of rapid growth, including in delivering new technologies. Centralized databases greatly enhance the utility of such data. In 2021, 19 y after its launch, eBird (50)—where people from around the world can submit bird observations—recorded its billionth observation. In 2025, this number surpassed two billion, collected by 1.1 million people. These enormous quantities of data have enabled mapping of species range limits, indices of abundance for almost 3,000 species, and trend estimates at spatial resolutions of 27 x 27 km for several hundred species (51). Similarly, by June 2025, iNaturalist had amassed almost 250 million observations of over 5,18,000 species, mostly with photographic documentation, contributed by more than 3.7 million people (https://www.inaturalist.org/).

Digitization of natural history collections, in museums and herbaria, have the potential to make accessible far more of the over three billion specimens worldwide linking historical collections with current observations (52). These records have a direct link to a physical voucher in a museum or herbarium and are collected by professional taxonomists/systematists; digitization of specimen images will also provide well-annotated data sources for AI-based species recognition.

There has also been considerable development of remote sensing technologies in recent years. Remote sensing now allows detailed mapping of ecosystems, for example, Global Forest Watch [(53) and coastal systems (54)]. Technology is continually improving, for example, with hyperspectral satellites and P-band radar there is an expanding frequency range and greater resolution and frequency of surveys and open access datasets, such as the European Space Agency’s Sentinel 2 that provides 10 m resolution data every 5 d. Alongside this enormous increase in data a promising avenue for improving the use of remotely sensed data comes from the development of geospatial foundation models, especially those that integrate data from multiple sources including optical, radar, LiDAR, and other sources, such as those within Google Earth Engine platform (https://earthengine.google.com). These systems have massive potential to monitor landcover and land-use changes, dynamics in tree species composition, map pressures, model species distribution and examine the consequences of conservation measures. These AI-enabled remote sensing could dramatically change biodiversity measurement. Earth observation data already help policy frameworks be measurable. For example, 41% of the Global Biodiversity Framework headline indicators are spatially explicit and reliant on earth observation (https://unbiodiversitylab.org/en/monitoring-framework-of-the-kunming- montreal-global-biodiversity-framework-data-collection/).

The development of low-cost wearable sensors means animal movement can be documented at scale, improving the quantification of dispersal, migration (55), behavior, physiology, health, and interactions of individuals. These high-resolution, widely distributed data have the potential to greatly improve ecological predictions, for example, the capacity of species distribution models to predict responses to climate change.

## Recommendations for Changing Biodiversity Measurement to Help Tackle the Biodiversity Crisis

We make nine recommendations for making the most of the rapidly changing landscape for biodiversity measurement to ensure it can contribute to tackling the biodiversity crisis.

### Capitalize on the Ability of Novel Technology to Integrate Data Sources.

Biodiversity information typically comprises data from a single different source, including auditory, eDNA, genomics, Indigenous Knowledge, museum specimens, remote sensing, and visual. A major current development is combining of different sources of information to create holistic models that capture both broad-scale patterns from satellite imagery and fine-scale, ecologically relevant information from ground-based measurements to produce a more comprehensive and accurate picture of global biodiversity (56–58). These complementary data sources offer scalable methods for biodiversity monitoring, providing granular insights that single methods alone cannot capture.

Advancements in AI, particularly generative AI that is hallucination-free, represent an opportunity to derive insights across these often disconnected data sources. Generative AI enhances our capacity to collect, process, and synthesize biodiversity data at scale by combining and analyzing existing datasets efficiently. These tools can reveal undiscovered patterns that would be difficult to achieve through manual synthesis alone. For instance, AI applications have been used to model species distributions more accurately, improve habitat classification, identify invasion pathways of alien species, and predict zones of human-wildlife conflict (32). They can also support the analysis of trade routes in illegal wildlife markets by uncovering hidden patterns in complex data.

The bottleneck for depending on insights from generative AI is a robust validation pipeline based on accurate ground truth, especially in spatial regions with historical data gaps. A serious primary challenge therefore lies in funding and implementing integrated monitoring efforts over sufficiently large regions or developing cost-effective methodologies that can be reliably extrapolated over larger geographic areas. Overcoming these logistical and financial hurdles is paramount to developing comprehensive and scalable solutions for global biodiversity and ecosystem condition assessment.

### Agree Standard Methods for Data Collection.

Inconsistency in methods for measuring biodiversity hinders all major uses of data: comparisons of global diversity, assessment of change, determining effectiveness of actions, and reporting on performance. For example, in a meta-analysis of phenological change, Brown et al. (59) found that methodological variation explained almost half as much variance as biological variables. More generally—for country, business, NGO and community use cases—there is a need for a holistic approach that provides a framework for multistakeholder global monitoring standards. Potentially coordinated by an international body, such a framework would develop, endorse, and promote a tiered and modular monitoring workflow—from data acquisition and management through to analysis, indicator calculation, and reporting (60). The framework would include existing standards for fundamental interoperability [e.g., the Darwin Core (61)] and more advanced, specific standards for various biomes, taxa, and technologies (e.g., eDNA, remote sensing, and the use of AI). Crucially, there is a need for clear guidance, capacity-building programs, and incentives (including linking to funding and regulatory requirements) to ensure widespread adoption. A monitoring standards framework [following FAIR and CARE principles, (62)] would ensure that data workflows are verifiable, discoverable, auditable, reusable, and ethically managed, particularly concerning Indigenous peoples and local communities’ knowledge.

This can build on the standards and lessons from a range of existing communities who have standardized processes, such as The US National Ecological Observatory Network (63), the US Forest Service Inventory Analysis (FIA), the grassroots global Nutrient Network (64), Forest GEO (65), and TreeDivNet (66). In forest ecology, standardized methodologies have been developed for establishing forest plots and monitoring the trees they contain [e.g., ref. 67], which has generated a global network of sites yielding long-term data enabling global analyses, greatly strengthened by consistent data. There may be lessons from the biodiversity genomics community, including The Earth BioGenome Project (68), who established an International Science Committee that created a set of standards, whose adoption contributed to the rapid growth of annotated, reference-quality genomes (40). Critical lessons from these precedents are the importance of early integration, collaborative global governance, scalable protocols adopted across institutions, and a robust data-sharing ethos encouraging data reuse and synthesis.

The goal of these efforts is to minimize the use of inconsistent methods where viable alternatives exist, while recognizing that standardization is not always appropriate—particularly in long-term monitoring where consistency is important. Methodological differences can also be justified by local conditions or evolving technologies. To address this, standards should offer guidance for comparing new and existing methods in an open and transparent way (discussed in greater detail in the following section). Their adoption depends on strong incentives, capacity building, and inclusive data governance. Ultimately, transforming data comparability, reliability, and utility is essential for evidence-based decisions and tracking progress toward the Global Biodiversity Framework’s 2,030 targets. The need for increasing standardization across the monitoring workflow and suggested options for implementing this is explored in much greater detail in a parallel paper (60).

### Ensure New Technologies Are Calibrated with Existing Data.

Despite the many exciting opportunities afforded by the new approaches outlined above, integrating data from multiple sources faces a range of challenges to ensure comparability. A major challenge with any change in observation methodology is that recorded differences in biodiversity could be due either to a genuine change or a methodological difference. This is especially the case for sensor and AI-generated biodiversity measures, highlighting dramatic changes in the observation process from traditional field surveys to autonomous and remote methods. While this presents a challenge for spatial comparisons (e.g., is a site especially rich or subject to a new method?), it poses a greater issue when evaluating changes over time. There is a need to ensure data are interoperable enough for spatial and temporal comparisons.

If the new and old schemes are not properly cross-calibrated, the change could confound long-term trends. Analytical frameworks to overcome this challenge of multiple data sources have developed rapidly (69), but their success relies on a clear and detailed documentation of observation metadata for each monitoring scheme, and ideally, the operation of both schemes at overlapping locations for some time to allow direct comparisons. For example, the parallel operation for a 6-y period of both the Common Birds Census and the Breeding Bird Survey in the United Kingdom allowed the old and new datasets to be combined while enabling coverage to increase more than 15-fold (70, 71).

Similar cross-comparisons will be particularly crucial when sequencing, or sensor-based approaches, provide a different biodiversity measure than existing schemes, for example, when monitoring switches from counting individual animals to counting vocalizations or other acoustic indices (72), or from traditional observational field surveys to eDNA (73). Archiving samples used for eDNA analyses and the raw data from such monitoring schemes can help facilitate reanalysis and understand the consequences of evolving hardware and software pipelines (74).

### Fill Data Gaps by Using Emerging Technologies and Increasing Capacity, Especially in the Tropics.

The geographic and taxonomic distribution of biodiversity data show profound and complex biases (e.g., refs. 75 and 76) that hamper the production of ready-to-use information on spatial patterns and temporal trends. The paucity of data from the tropics, soils, mesopelagic zones, and deep ocean—all rich in unique biodiversity and all facing threats from human actions—places such systems at a disadvantage when decisions are made that affect them (77).

The wealth of data for a few charismatic taxa [most obviously birds, which dominate the largest global biodiversity database, GBIF (78)] facilitates conservation decisions tailored to those groups, but these choices may not be optimal for other groups with different distributions [e.g., Insects (79)]. Invertebrates are particularly underrepresented in biodiversity conservation thinking. So are undescribed species (“dark biodiversity”), many of which are likely to be endemic insects. Soil biota and microorganisms are even less well understood.

Beyond taxonomic groups, geographic gaps are evident, especially in the tropics where, as well as a shortage of trained people and equipment, there is also usually little eDNA reference material or auditory training material. The need for increased capacity in the tropics is a clear priority. As a result of these gaps and biases, conservation goals, targets, and policies are set based on information from species that are much more widely distributed and much more ecologically generalist than most of Earth’s species.

### Create Living Databases of Trusted Information to Reduce the Risk of Poisoning by AI Hallucinated, or False, Information.

There is long history of fraudulent specimens, including the creation of new species [Piltdown man (80)], importing birds and claiming found in Britain [The Hasting’s rarities; (81)], stealing bird specimens from collections and relabeling [Colonel Meinertzhagen (82)]. Similarly, there is an increasing problem of fictitious research papers, including those created by paper mills. AI algorithms are known to generate plausible but fictitious details, known as AI hallucination. A second issue with AI is its role in facilitating the creation of ever larger numbers of convincing, but fabricated, data. These could be through AI-generated images for image-based databases of field observations (or auditory equivalents) or through the creation of fabricated papers with invented data. The novel challenge is that with AI, this fabrication can be achieved easily and at scale. The motives include career gain, being disruptive or pushing an agenda (for example, by making an “at risk” species threatened by a project seem more common or purporting to show biodiversity gains, or no harms, in previous projects). This represents an existential crisis for AI data analysis and evidence Synthesis (83).

Current processes seem poorly equipped to deal with this problem. Gold standard systematic reviews are resource-intensive, yet many already unwittingly cite retracted publications (84), with 89% remaining uncorrected a year after being notified of retractions (85), this problem will be exacerbated by an increasing volume of AI-fabricated material. Rather than depending on the individuals analyzing information to judge validity a suggested better approach for the literature is an institutionally federated network of living evidence (i.e., continuously updated) databases to ensure the scientific integrity of the results.

Dynamic, specialized hallucination-free AI systems could continuously gather, screen, and index literature relevant to defined themes and its measured outcomes, automatically tagging compromised studies for scholarly review. This could provide a robust, transparent, and dynamic source of scientific knowledge, enabling high-quality systematic reviews. Rapid reviews could be undertaken and updated in real time through processes such as dynamic meta-analysis (86). This approach could safeguard evidence synthesis against the rising tide of potentially poisoned literature (83). For biodiversity data, a similar process of active vetting of databases is likely to be required.

### Ensure Data Generation Is Valued.

The process of collecting biodiversity data is often difficult and time-consuming and must not be undervalued. While some new technologies may reduce reliance on field-based data collection, there remains a crucial role for locally based expertise. Even satellite-mounted sensors recording changes in habitat extent or condition require fine-scale ground-truthing drawing on local field effort. Systems to support citizen scientists require investment as coordination is crucial. While there are increasing calls for all biodiversity data to be public (87), making all data publicly accessible could lead to a producer-scrounger model, as without appropriate incentives, it pays to use data rather than generate it. Despite widespread acceptance of the importance of open science (88), data underpinning scientific publications, many journal articles, including in ecology, still fail to make data available (89), however, change is underway. The lessons learned from the experience of efforts to increase data sharing in ecology have been the following: i) There needs to be governance for wholesale change, in this case through the journals and societies involved; ii) credit for reuse of data should be given, through coauthorship and citable sources, such as digital object identifiers (DOI), alleviating concerns over data ownership and credit; iii) the production of data can be regarded as a creditable output in itself, e.g., through recognition by grant bodies and in research assessment exercises; iv) there needs to be sufficient resourcing, in this case through direct funding or contributions of learned societies to archiving costs (e.g., the British Ecological Society covers archiving costs of papers published in its seven journals).

Many of the classic early descriptions of tropical biodiversity attributed to European explorers, such as von Humboldt, Darwin, and Wallace, relied heavily on local and Indigenous assistants to find, collect, and classify the specimens (90). Emogor et al. describe a process for giving extended credit when it is inappropriate or against the journal’s policy to include contributors as authors (91).

### Ensure Respectful Incorporation of Indigenous Knowledge.

Indigenous Knowledge (IK) is a collective term representing the many place-based knowledges, innovations, and practices accumulated across generations through interaction with the environment (92, 93). While each Indigenous community has a unique culture, so definitions and terminology may vary, these knowledge systems are inseparable from their culture’s spiritual and social fabric and incorporate moral values, such as kinship with nature, humility, and reciprocity (94–96).

Indigenous communities have felt pressure by colonizers to assimilate, which has led to the loss of medical, ecological, and cultural information (97). Given the history of forced assimilation, it is important that indigenous knowledge is collated and shared by Indigenous peoples themselves and on their own terms (98). The Pisuna program in the Arctic is an example of a successful community-based monitoring program documenting their knowledge in a culturally appropriate manner (99). The development of AI risks widening the gap between Indigenous peoples and scientists, although there are good examples of AI being used with Indigenous Knowledge to inform decision-making (100).

The value of indigenous and local knowledge is increasingly recognized in science policy arenas such as Intergovernmental Panel on Biodiversity and Ecosystem Services and the Convention of Biological Diversity (101). For example, the Global Biodiversity Framework’s “monitoring framework” provides pathways for indigenous knowledge to be incorporated synergistically. Indigenous voices are also increasingly being heard in these international agreements—for example, through the International Indigenous Forum on Biodiversity (IIFB) under the CBD (https://iifb-indigenous.org/about-us/). Such initiatives are crucial both for moral reasons, and practical reasons given the role of Indigenous peoples as steward of biodiversity (102).

### Ensure Measurements Enable the Quantification of Effectiveness of Actions.

Monitoring biodiversity outcomes without a focus on designs which allow insights into the impact of actions to address the biodiversity crisis has been referred to as “counting the books while the library burns” (103). Such an approach can only describe declines, rather than help address the biodiversity crisis directly. There is therefore an explosion of interest in measuring the effectiveness of conservation policies and practices (37) to inform truly evidence-based policy making (35).

New technologies can help provide the biodiversity data at sufficiently high spatial and temporal resolution needed to estimate the counterfactual: What would have happened without the action (104). However, large amounts of data do not overcome the need for study designs that allow causation to be separated from correlation. Embedding impact evaluation into conservation action at a much larger scale is therefore needed (36, 105).

### Increase the Resilience of Global Datasets to Technical and Societal Change.

Challenges to maintaining information are not new, as illustrated by the destruction of the grand library of Baghdad in 1258. Recent events, including the fire in the National Museum of Brazil in 2018 destroying most of the 20 million items, the cyberattack attack on the British Library, and the closure of key herbaria (e.g., Duke) and museums that have been home to precious collections, illustrate the potential damage that could be done to biological collections and data. We note with particular concern actions taken by the US government to close US datasets, including more than 2,000 from data.gov (106).

Another issue is data storage that uses proprietary software, since all software eventually becomes obsolete. Data should always also be stored in universal formats, such as comma separated data files with associated text metadata. Solutions include ensuring data backups in resilient forms, multiple funding sources such as public–private partnerships to reduce the reliance on single sources, dispersed systems with multiple versions, geopolitically diverse hosting to reduce the impact of political change, and, where appropriate, condemnation by society and global researchers. An important goal is to incorporate biodiversity data into a nationally or internationally mandated system with explicit backup and resilience mechanisms, as is provided for DNA sequence data by the International Nucleotide Sequence Database Collaboration (INSDC; linking GenBank, the European Nucleotide Archive and the DNA Database of Japan).

A comprehensive deployment of sensors for biodiversity measurements could easily encompass tens of millions of devices being deployed in remote areas of the planet, which need to be networked together to collate the information. There are similar concerns with the resilience of the Internet itself as the long-term mesh for this. The global network is expected to expand to a trillion nodes this decade but faces a range of challenges from large quantities of fake, generative, or poor data, sophisticated AI-driven malware, botnets commandeering huge numbers of machines to launch attacks, and even alteration of the physical world through the manipulation of Internet of Things (IoT) devices. Madhavapeddy et al. (in press) explore how the core Internet architecture might draw from ecological theory to find means of ensuring it remains a resilient, sustainable, and trustworthy network, which is vital given our growing dependency on digital measurements of global biodiversity (107). Ideas for a biologically inspired resilient Internet include ensuring greater diversity in the software stacks that comprise Internet nodes, active response to challenges (much like antibodies respond to infections), and providing shielding to hosts unable to deploy countermeasures. All these approaches will help ensure the security and integrity of primary biodiversity measurements over longer periods of time.

### Final Thoughts.

Biodiversity scientists are on the cusp of a transformative flood of new data, due to the combination of increasing demands for ecological information, more comprehensive and sophisticated analyses, and astonishing technological developments. Here, we identify a set of recommendations which will make the most of these opportunities while addressing the challenges. Delivering on these will require novel collaborations between communities who have not traditionally collaborated closely. Computer scientists, engineers, molecular biologists, data scientists, field ecologists, citizen scientists, Indigenous peoples, policy makers, and local communities need to work together to create rigorous, resilient, and accessible biodiversity information systems. The ultimate aim is to deliver real-time, localized but globally scalable assessments of biodiversity dynamics to inform decision-making by diverse stakeholders at the temporal and spatial scales that are needed.

## Data Availability

There are no data underlying this work.
